# Expanding reimbursement of immediate treatment using direct acting antivirals to reduce hepatitis C incidence among HIV positive men who have sex with men in Bangkok, Thailand: A cost effectiveness modelling study

**DOI:** 10.1016/j.jve.2021.100042

**Published:** 2021-05-18

**Authors:** Shreoshee Mukherjee, Donn Colby, Reshmie Ramautarsing, Stephanie Popping, Somchai Sriplienchan, Tanat Chinbunchorn, Nittaya Phanuphak, David van de Vijver

**Affiliations:** a Erasmus MC, Department of Viroscience, Rotterdam, the Netherlands; b United States Military HIV Research Program, Walter Reed Army Institute of Research, Silver Spring, MD, USA; c The Henry M. Jackson Foundation for the Advancement of Military Medicine, Bethesda, MD, USA; d Institute of HIV Research and Innovation, Bangkok, Thailand; e Department of Medical Microbiology and Infectious Diseases, Erasmus MC, Rotterdam, the Netherlands

**Keywords:** MSM, Cost effectiveness, Hepatitis C, HIV, Direct acting antivirals, Micro elimination, Thailand, Asia

## Abstract

**Background:**

Increasing number of hepatitis C virus (HCV) infections among HIV positive men whohave sex with men (MSM) as in an acute HIV infection cohort study in Bangkok, reached an incidence of 45/1000 person-years in 2018. Direct-acting antivirals (DAAs), that cure HCV infection and thereby can prevent transmission, are expensive, their reimbursement being presently delayed to the chronic stages of liver fibrosis. The aim of this study was to determine the cost-effectiveness of immediate DAA treatment to reduce HCV transmission among HIV positive MSM in Bangkok.

**Methods:**

A deterministic transmission model was calibrated to the HCV epidemic among HIV positive MSM in Bangkok. We compared the current practice of starting DAAs at METAVIR stage F2 rather than at stage F1, or immediately after diagnosis, at stage F0. Cost-effectiveness was examined from a payer's perspective, using a 3% annual discounting rate.

**Results:**

Compared to the incidence in 2018, delaying DAA treatment to METAVIR stage F2 or F1, increases HCV incidence in 2030 to 63/1000 person-years and 56/1000 person-years, respectively. Conversely, immediate DAA treatment reduces the incidence to 26/1000 person-years. Compared to initiating treatment at stage F2, immediate treatment is cost saving within seven years and saves $17 million over 40 years. One-way sensitivity analysis showed that lower cost savings were achieved at a higher price of DAA treatment and at less frequent HCV screening.

**Conclusion:**

Immediate DAA treatment is cost saving and increases health benefits by reducing HCV incidence among HIV-infected MSM.

## Introduction

Hepatitis C virus (HCV) is a major global health issue, affecting an estimated 71 million individuals globally.^1^ The introduction of direct-acting antiviral (DAA) drugs in 2014 has been a breakthrough in the treatment of HCV with more than 95% cure rates.^2^ DAAs have also improved prevention, as cured individuals cannot transmit the virus to others.^3^^,^^4^ Recognizing this breakthrough, the World Health Organization declared in 2017 the ambitious goal of eliminating HCV transmission by 2030.^5^

In Bangkok, an emerging HCV epidemic has been observed among HIV positive men who havesexwithmen (MSM). As part of the SEARCH 010/RV254 acute HIV cohort study based in Bangkok, HIV positive MSM were followed up routinely and screened for HCV infection during their HIV clinical care. Although no new HCV infections were observed before 2014, the incidence increased to 45 per 1000 person years by 2018^6^.

According to the latest Thailand Practise Guideline to Elimination of Hepatitis C published on May 2, 2020,^7^ DAAs are recommended irrespective of the stage of fibrosis. However, as per the National Health Security Office reimbursement criteria, DAAs are restricted to the more advanced stages of fibrosis by only reimbursing DAAs for METAVIR stage F2 and higher of liver fibrosis. Additionally, as per our guidelines, HCV screening is conducted annually only among HIV positive people who inject drugs (PWID). Given the increase in incidence among HIV positive MSM, baseline and annual testing for HCV could allow for improved diagnosis. Subsequently, reimbursement of DAA treatment unrestricted by the stage of liver fibrosis could enhance DAA uptake, resulting in health benefits and reduced HCV transmission. In this study, we aim to assess whether the costs of expanding DAA reimbursement in Thailand for genotype-1a in HIV/HCV coinfected MSM is affordable for the Thai government using epidemiological, cost-effectiveness and budget impact of DAA treatment analysis.

## Methods

### Deterministic transmission model

A previously published Dutch deterministic HCV transmission model^8^ was calibrated to represent the Thai HCV epidemic among HIV-infected MSM from 2015 to 2018. The emerging HCV epidemic has been well described by the SEARCH 010/RV254 research team in Bangkok that collects data on all relevant parameters such as the number of new HCV infections^6^ to analyse epidemiological trends, HCV phylogenetic distribution, route of transmission, cost of treatment and economic impact.

### Model assumptions and calibration

The Dutch model was adapted to represent the Thai HCV epidemic amongst HIV positive MSM (Table 1 and supplement). We calibrated our model to the historic HCV epidemic using the estimated Bangkok MSM population size (120,000 to 250,000)^9^ with a 5-year growth rate (2.31%), proportion of MSM infected with HIV (19.5%),^10^ proportion of HIV positive MSM diagnosed with HCV (8.7%),^11^ rate of new HCV infections (32.3 per 1000 person years in 2017, 44.8 per 1000 person years in 2018),^6^ and rate of HCV reinfection of 14.1% [5%–19%]^12^ after treatment or spontaneous clearance. Using Monte Carlo filtering technique, we accepted 128 simulations of 100,000 as per the calibration range that represented the Thai HCV epidemic among the HIV positive MSM. We included variables such as duration of treatment, the population of HCV/HIV co-infected MSM in the year 2013, when the model is seeded.

**Table 1:** Modelling parameters.

Sampled Parameter	Value [range]	Source
**Entry rate of HIV-infected MSM**	Varied over annual sexual risk activity e.g., number of new sexual partners	Calibrated
	C1: active [>20,100]	
	C2: number of new sexual partners every year upper-medium active [>5, 20]	
	C3: number of new sexual partners every two years medium active [1, 5]	
	C4: number of new sexual partners every two years least active [< 1]	
**Proportion of MSM in risk groups**	P1: highest risk group [0.01, 0.15]	Calibrated
	P2: upper medium risk group [0, 0.2]	
	P3: medium risk group [0, 0.3]	
	P4: proportion in low risk group [1 – (p1+p2+p3)]	
**Year of HCV epidemic seeding**	2013	Calibrated
**Life expectancy of Thai men at birth (total years)**	73 years	^26^
**Mortality**	1/45	^27^
**Proportion of HIV-positive who spontaneously clear acute HCV infection**	15–20%	^13^^,^^28^^,^^29^
**Duration until spontaneous clearance**	40–170 days	^29^
**SVR with DAA**		
**Fibrosis**	98.5% [95–100%]	^2^
**Compensated cirrhosis**	[80–95%]	
**Decompensated cirrhosis**	[70–80%]	
**Duration of treatment**		
Fibrosis	12 weeks	^30^^,^^31^
Cirrhosis	24 weeks	
**Quality Adjusted Life Years (QALYs)**		
QALYs when only HIV positive	0.94	^32^^,^^33^
QALYs in F0	0.89*0.94	
QALYs in F1	0.89*0.94	
QALYs in F2	0.89*0.94	
QALYs in F3	0.89*0.94	
**QALYs in compensated cirrhosis**	0.38 + 0.5*0.29	
**QALYs in decompensated cirrhosis**	0.38	
**QALYs in hepatocellular carcinoma**	0.45	
**Parameters used for model fitting (point estimate, range)**		

**HCV incidence among diagnosed HIV-positive MSM**		
2017	32.3 per 1000 [22–43]	
2018	44.8 per 1000 [35–55]	^6^
**HCV prevalence among diagnosed HIV-positive MSM**	8.7% [7.5–10%]	^11^
**HCV reinfection rate after treatment or spontaneous clearance**	14.1% [9–19%]	^12^
**Number of MSM in 2015**	185,000 [120000–250000]	^9^
**HIV Prevalence among MSM**	19.5% (23000–49000)	^34^

The model stratifies into those individuals who were not infected, those infected, and those who have been cured but susceptible to reinfection. Further, infection with HCV was stratified by patients undergoing spontaneous clearance (15–20%)^13^ or moving into three progressive stages of fibrosis (METAVIR stage F0, F1, F2, F3), two stages of cirrhosis (F4; compensated cirrhosis and decompensated cirrhosis) which in turn could lead to development of hepatocellular carcinoma. At each stage of disease progression, the model was compartmentalized into individuals undergoing treatment with DAAs and those who did not achieve a sustained virological response after treatment. The schematic representation of the model along with the equations can be found in Fig. S1 of the supplement.

In the baseline scenario, individuals start treatment with DAAs at METAVIR stage F2, since they are reimbursed at this stage as per the Thai hepatitis C management guidelines.^7^ The base case is then compared to two scenarios in which a) DAAs were prescribed at METAVIR stage F1 and, b) immediately after diagnosis. Duration of treatment was 12 or 24 weeks, for treatment in stage F0 through F3 or people who had cirrhosis, respectively. The probability of achieving SVR varies between 95 and 100% in stage F0 through F3 of fibrosis, and 80–90% for cirrhosis.^2^

We took quality-adjusted life-year (QALY) estimates depending upon the model compartment or the stage of disease progression (Table 1). All results predicting prevalence, incidence, number of infections averted, cost incurred, health utilities with respect to quality-adjusted life-years (QALYs) gained, are reported as median and interquartile range (IQR) of the accepted simulations.

### Cost-effectiveness methods to recognize optimal treatment scenario

The costeffectiveness analysis used a payer's perspective over a lifetime, a 40-year time horizon, at 3% annual discounting. Costs were valued in 2020 US dollars (USD) (1 Thai Baht = 0.032 USD) and health outcomes were valued in QALY. Costs of drugs and diagnostic tests were used as per the Thai Hepatitis C Management Guidelines 2018 which delineates the associated protocols during screening, diagnosis, and treatment.^14^ The associated costs at each stage or compartment of the HCV transmission mathematical model correspond to the diagnostic tests and drugs used in that compartment, extracted from centralized regional public hospitals by the SEARCH 010/RV254 research team. The costs of the DAA drugs were taken for a combination of sofosbuvir/ledipasvir used to treat HCV-infected patients with the genotype 1.

The median and interquartile range of the costs and QALYs were tabulated for each accepted simulation at every stage of fibrosis. Thereafter, increment in costs and QALYs were calculated. The incremental cost-effectiveness ratio (ICER) for the intervention scenarios was then evaluated to make the cost- effectiveness table. The intervention with the highest ICER was selected as the most cost-effective intervention while other scenarios were deemed to be dominated. We also examined the short-term budget impact over 10 years on undiscounted costs cumulated for immediate and early-stage treatment intervention.

### One-way sensitivity analysis

We performed a one-way sensitivity analysis to assess the impact of uncertainty in the costs of drugs and diagnostic parameters on the incremental cost-effectiveness ratio over 40 years for immediate DAA treatment. We examined how sensitive the modelled cost-effectiveness was to changes in the input cost variables. The cost of 12-week regimen of DAA-based drug combination sofosbuvir/ledipasvir in Thailand is 13,860 THB or 445.8 USD, which was varied to upper and lower limits over a range of 50%. The upper limit was considered as 20,790 THB or 668 USD while the lower limit was considered as 6930 THB or 222.9 USD. We varied the costs of the rest of the diagnostic tests as per the range of the cost as charged in public hospitals. We also varied the frequency of HCV RNA tests conducted from 6 months to 1 year and 3 years.

We performed a multivariate sensitivity analysis using recursive partitioning to determine the most influential independent parameters on the cost-effectiveness of the DAA-based treatment of HCV. The impact of parameters like the incidence rate, prevalence, reinfection rate was observed on the cost- effectiveness of immediate treatment as compared to the base case scenario.

## Results

**Fig. 1:**
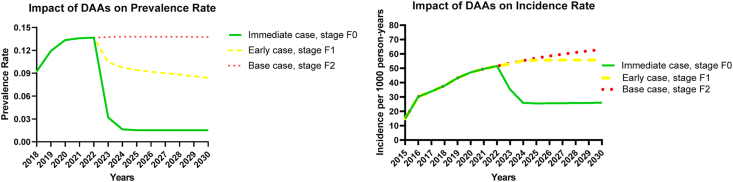
a) Impact of Direct Acting Antivirals (DAAs) on the rate of prevalence at the three intervention scenarios; Fig. 1b) Impact of DAAs on the rate of incidence per 1000 person years of follow-up from the beginning of the epidemic up until 2030.

Our model projects that continuing delaying DAAs to METAVIR stage F2 of liver fibrosis, increases the incidence rate of HCV infection from 45 per 1000 person-years in 2018 to 63 per 1000 person-years of follow-up in 2030 among HIV-infected MSM. If DAAs are started in stage F1, then the increase in incidence is relatively lower, going up to 56 per 1000 person years of follow-up in 2030. Immediate treatment with DAAs upon diagnosis has a profound preventative impact on HCV infection as indicated by a reduction in incidence rate to 26 per 1000 person-years of follow-up in 2030 (Fig. 1).

**Fig. 2:**
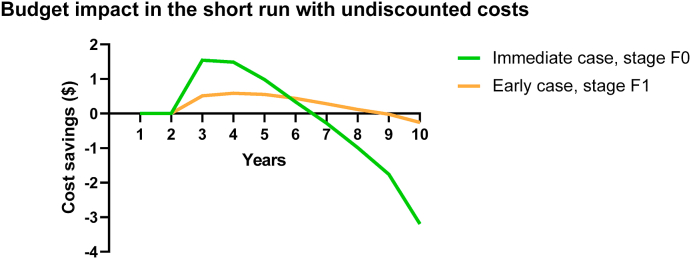
Using undiscounted costs, the impact on the budget when immediate DAA treatment is introduced after diagnosis; and when treatment is initiated to early METAVIR stage F1 of liver fibrosis. Break-even point is reached within 7 years when treatment is started immediately after diagnosis.

Continuing the current practice of delaying treatment to fibrosis stage F2 will have a profound impact on the HCV prevalence among HIV positive MSM in Thailand, which will increase from 8.7% in 2018 to 13.7% in 2030. Starting treatment earlier at stage F1 will result in a prevalence of 8.3% in 2030. However, the prevalence is predicted to dramatically decrease by over 80% if the treatment is initiated immediately upon diagnosis, reducing to 1.5% in 2030 (Fig. 2).

**Fig. 3:**
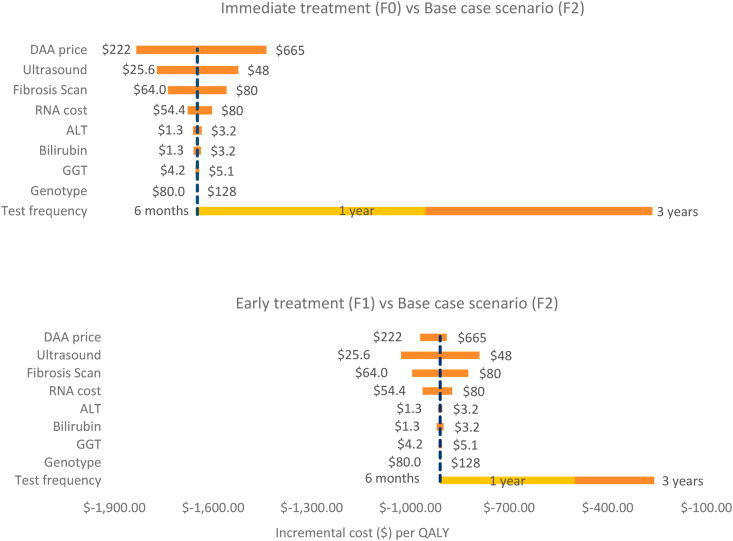
One-way sensitivity analysis to show the variation in the prices of drugs and diagnostic tests on cost-savings.

The strong impact of immediate treatment is also reflected in the number of HCV infections averted until 2030 among HIV-infected MSM. With the treatment being initiated at F1 stage rather than the delayed F2 stage of fibrosis, only 474 new infections are predicted to be averted through 2030, whereas 5939 new infections are averted if the DAA-based treatment is initiated immediately upon diagnosis rather than F2 stage of liver fibrosis (Fig. 3).

The discounted cumulative costs of DAA treatment of HIV-infected MSM will be 46 million USD [IQR: 35.3–51.6] in Thailand if DAAs are introduced at stage F2. Compared to the base case scenario, advancing the treatment to F1 stage will lead to economic gains, saving 5 million USD [IQR: 4.2–5.8]. Importantly, early treatment at F0 stage of fibrosis would save substantial costs worth 17 million USD [IQR: 14.3–18.8] over the lifetime period of 40 years. Similarly, the quality-adjusted life years (QALYs) are observed to increase with an earlier treatment stage from the delayed F2 stage (900,000) to the chronic F1 stage (903,000) and finally to the early acute F0 stage (906,000) of liver fibrosis as the number of new infections averted improve with expansion of treatment. Thus, the incremental cost-effectiveness ratio is inferred for stage F0 to be the most cost-effective stage of intervention while the chronic F1 stage and delayed chronic F2 stages of liver fibrosis are dominated, as shown in Table 2.

**Table 2:** Cost-effectiveness table comparing the three scenarios at which Direct Acting Antivirals are introduced in Thailand in the model.

Scenario	Total Costs, USD (millions)	QALY *1000	Incremental Costs USD (millions)	Incremental QALY	Incremental Cost Effectiveness Ratio	Cost effectiveness
**Immediate case, stage F0**	29	906	−17	6000	2833	Most cost saving
**Early case, stage F1**	41	903	−5	3000	5555	Dominated
**Base case, stage F2 stage**	46	900	NA	NA	NA	Dominated

Fig. 2 shows that expanding the DAA-based treatment to the early acute F0 stage of fibrosis would be initially expensive because the HIV-infected MSM population under treatment coverage will go up. However, within seven years, we observe that the costs saved from averting new infections by expanding treatment from F2 stage to F0 stage exceeded the costs incurred in expanding the treatment to early acute stage F0 of liver fibrosis. Therefore, within seven years, the investment on expanding the DAA-based treatment to the early stages of fibrosis breaks even and is recovered from the costs forgone by averting new infections.

The one-way sensitivity analysis in Fig. 3 shows the impact of varying the costs of drugs and diagnostic tests on the cost-effectiveness ratio. The model predicted that the cost of DAAs has the strongest impact on cost savings as compared to other cost inputs when treatment is started immediately in F0 as compared to the base case F2. Testing for HCV at a 6 months' interval is more cost saving than testing once a year or once in 3 years. As can be seen in Fig. 3, changing the price of ultrasound and fibrosis scan costs have an impact on cost savings, whereas HCV genotyping has no impact on cost savings. Further, we conducted multivariate sensitivity analysis which showed that the magnitude of the incidence rate in the year 2030 had a dominant impact on cost-effectiveness. This could be ascribed to the fact that the cost of elimination can have a U shape curve. Initial costs of investments into expanding treatment are high, which progressively are reduced, only to become high again. Finding the last infected individuals in the population is expensive. Other variables that were also significantly impacting the cost-effectiveness were percentages of reinfections, number of people diagnosed with acute HCV infection, proportion of people transitioning from delayed stage 4 of fibrosis to compensated cirrhosis, and proportion of people in the highest sexual risk activity class [Suppl. Fig. 2].

## Discussion

Our model predicts that introducing DAAs immediately after HCV diagnosis among HIV-infected MSM is cost saving, i.e., reduced costs with gain in QALYs, which kicks in within a short timespan of 7 years of investment. For the first seven years, the undiscounted costs of immediate DAA treatment are higher than the current practice of delaying treatment to METAVIR stage F2 of liver fibrosis. After seven years, cost savings begin and can lead to a 42% reduced incidence rate, 80% reduced prevalence, and 12 times more infections averted through 2030 with immediate treatment. These results can encourage policy makers to invest in reimbursing immediate DAA treatment to promote reduced HCV burden within HIV-positive MSM.

Micro-elimination efforts have been frequently discussed as a strategy of HCV elimination among key populations that predominantly transmit the virus.^15^ HIV/HCV co-infected MSM would be an ideal target group for these efforts as this is a well-defined risk group that actively contributes to new HCV infections in Bangkok. If these men are regularly screened for HCV during HIV routine clinical care, they can be diagnosed early and can start immediate treatment to prevent onward transmission. Although our study has shown that immediate DAA treatment can reduce the HCV incidence among HIV-infected MSM, micro-elimination is not achieved in 2030. Therefore, additional measures, including needle exchange programs, more frequent HCV screening and risk reduction programs must be considered to achieve the ambitious goal of ending HCV transmission by 2030.

Increasing testing to screen PrEP users for HCV infection could be a useful strategy for an early diagnosis of HCV-infected individuals, who may otherwise remain undiagnosed. PrEP is recommended to be used by MSM at high risk of HIV infection. However, PrEP medication sharing amongst MSM at risk has been reported.^16^ Consequently, MSM may start PrEP in informal settings without regularly being screened for HCV infection. Such informal use may facilitate HCV transmission as new infections are not diagnosed timely and subsequently remain uncured. Although the limited available information suggests that HCV is not frequently found amongst PrEP users, a surge cannot be ruled out. Therefore, micro-elimination of HCV transmission amongst MSM may in the future be targeted to all MSM at high risk of infection.

A direct policy implication of our study is that the cost of genotyping did not have any impact on the overall cost-effectiveness of immediate DAA treatment as per our sensitivity analysis. This is relevant since in the latest Thailand Practice Guideline for Eliminate Hepatitis C, 2020,^7^ genotyping is not required for pan-genotypic DAAs. In November 2020, sofosbuvir/velpatasvir (pan genotypic DAAs) became available in the National Health Security Office system and so genotyping is not required and will not be reimbursed. Since the impact of genotyping on cost-effectiveness is negligible, the revised policy guidelines are supported by our study results.

Several modelling studies from Asia share similar results showing that DAAs are cost-effective over a lifetime from a payer's perspective, with a few exceptions. The studies report costs, QALYs, ICER, as per the standard protocol that is followed in reporting cost-effectiveness modelling studies. The differences noted compared to our study are that firstly, other studies have used a Markov model, which contrary to our approach, does not account for the prevention of onward HCV transmission by DAAs, thereby underestimating the epidemiological impact. Secondly, these studies do not target the HIV positive MSM population, and consequently do not have any implication on micro-elimination strategies. Thirdly, the cost-effectiveness studies conducted in other Asian countries are from Hong Kong,^17^^,^^18^ Japan,^19^ and Singapore,^20^ which are high-income countries and therefore not comparable to Thailand. Among other comparable low- and middle-income resource settings, China^21^ did not find it cost-effective to use DAAs; a study conducted in Vietnam^22^ found DAAs cost-effective but took interferon-based treatments as a comparison, while another study in India^23^ uses no DAAs as a comparison scenario while concluding that they are cost-effective. Our study compares intervention scenarios of treatment using the same drug combination, sofosbuvir/ledipasvir, introduced at an early stage (immediately after diagnosis, stage F0), late stage (METAVIR stage F1), and delayed chronic stages of liver fibrosis (METAVIR stage F2); and finds it cost saving at all stages yet most cost-effective intervention is immediately after diagnosis of HCV.

There are several limitations to our study. First, the Bangkok epidemic is not well characterized with respect to reinfection rate estimates. We calibrated our model by using a range of reinfection rates and considering a point estimate from a Taiwan-based study.^12^ Conducting a multivariate sensitivity analysis using recursive partitioning, we accounted for the uncertainty of reinfection rates and showed that it has a dominant impact on cost-effectiveness outcomes. The implications of this finding are significant as future research is required to be conducted in the Thai context to get empirical estimates of reinfection among those cured of HCV after having been treated with DAAs. Second, the modelling has not been conducted from a societal perspective but from a payer's perspective. MSM who are uninfected with HCV would be healthier and reported ill less often when compared to HCV-infected MSM. There is evidence showing a loss in productivity^24^ from individuals infected with HCV or going through the process of recovery from the disease. This could lead to wages foregone and extra societal costs. Therefore, our current cost-effectiveness estimates are conservative. Taking societal costs into account would make the impact of interventions even more cost-saving. Third, the model makes assumptions considering a wide range of values on sexual behaviour as data is not available to build precise estimates on the sexual interactions between HIV positive MSM in Thailand. Irrespective of the wide ranges considered, all the results on DAA-based interventions are cost-saving because of the strong impact of these drugs on the incidence rate. So, although the assumptions fit the model, in future research contact tracing through phylogenetic studies could be added in the model to further narrow down the range. Fourth, our results are only applicable to HCV transmission in HIV positive MSM. We did not consider people who inject drugs (PWID), which is the largest risk group for HCV transmission in Thailand, because there is no systematically collected data on the incidence of HCV available in this population. Phylogenetic studies in the SEARCH study cohort have shown that Thai PWID and HIV positive MSM are infected with different genotypes. The genotypic distribution of the emerging HCV epidemic among HIV positive MSM was subtype 1a, as compared to genotype 3 in PWID. PWID and HIV positive MSM are two key populations where the HCV disease burden is concentrated. The prevalence of different genotypes indicates independent HCV transmission routes, as highlighted by a recent phylogenetic study that found that 85%^25^ of new HCV infections are found in clusters of HIV-positive MSM. Consequently, reducing the incidence through immediate DAA use in HIV positive MSM will not have an impact on the Thai HCV epidemic among PWID.

In conclusion, reducing the HCV disease burden among HIV positive MSM in Bangkok is most cost-saving when DAAs are introduced early as treatment and prevention strategy to reduce onward transmission. The Thai government investment in expanding DAAs to immediately after diagnosis, would contribute to the WHO's ambitious HCV elimination goals, but additional steps are required to eliminate HCV. An important consideration for governments is to examine the effects of any health investment in relation to their costs. Even if complete elimination is not possible, reducing the disease burden at a population level would be a priority for governments to be able to align with the WHO HCV elimination targets of 2030. To that extent, our study provides cost-effectiveness evidence on managing the disease amongst HIV positive MSM, a high-risk key population group,by immediately introducing DAA treatment. In its absence, rates of incidence and prevalence would keep increasing by 2030 leading to a huge cost burdento the Thai government. The evidence on substantial health gains and associated cost savings builds a strong case for the Thai government to expand reimbursement of immediate DAA treatment for individuals diagnosed with hepatitis C.

## Declaration of competing interest

The authors declare that they have no known competing financial interests or personal relationships that could have appeared to influence the work reported in this paper.
