# Supplementary material for: Expanding reimbursement of immediate treatment using direct acting antivirals to reduce hepatitis C incidence among HIV positive men who have sex with men in Bangkok, Thailand: A cost effectiveness modelling study
Source: J Virus Erad. 2021 May 18;7(2):100042. doi: 10.1016/j.jve.2021.100042 (PMC8184647; doi:10.1016/j.jve.2021.100042)

# Supplementary Information

**1. Hepatitis C epidemic simulation model**

Our simulation model follows a deterministic compartmental model for transmission, progression, and treatment; an approach generally used in HCV/HIV modelling. The schematic representation of the model has been presented in figure S1. The compartmental HCV model begins with (Ni), non-infected HCV individuals who are HIV infected MSM sub-divided into four sexual risk activity class (very-high risk, high risk, medium-risk and low-risk) which varies their probability of being susceptible to the virus (Si). Thereon, the individuals can either naturally clear the virus (Cl) and move to being susceptible to reinfection (Ri); or they can progress to acute stage fibrosis (F0). Within the fibrosis stage, the individuals can move into treatment (F0 Rx) where in if they achieve sustained viral response, they can move out of the model back to being susceptible to reinfection. If the individual fails treatment (F0 Fl), then they progress towards the next stage in severity of fibrosis (F1, F2, F3) and the same process continues as they move into compensated and decompensated cirrhosis (F4, F5) and finally develop hepatocellular carcinoma (HCC). In total, there are 10 states, out of which 9 represent HCV positive status (all but Ni). The total number of compartments are 22, the HCV negative, those susceptible, undergoing clearance, those susceptible to reinfection, three stages of fibrosis compartmentalized with treatment or failure; two stages of cirrhosis compartmentalized with treatment or failure leading to liver cancer.

**Figure S1:**


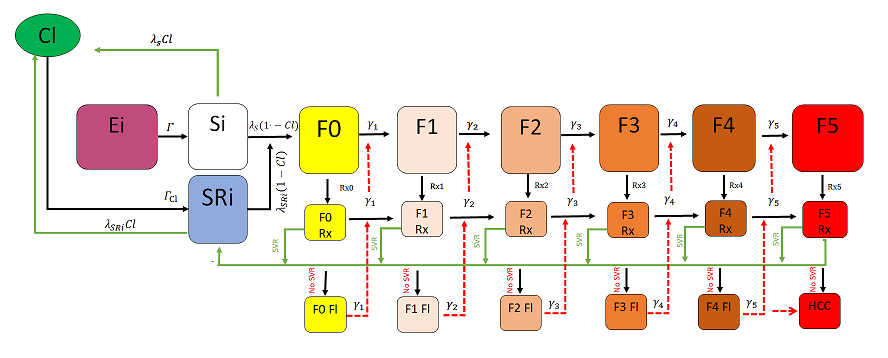


**2. Model Equations**

The model consists of six ordinary differential equations that represent the rate at which people are infected, or they clear the virus, or progress through stages of fibrosis. One equation describes mixing between individuals of different sexual risk groups at the entry point of the model. Two equations describe the force of infection or the rate by which individuals become infected and reinfected, having been susceptible to Hepatitis C. One equation describes the probability of clearing the virus naturally. Three equations describe the disease progression through different stages of fibrosis, cirrhosis, and hepatocellular carcinoma. The equations describe individuals detected at the stage of disease progression, when individuals are introduced to treatment in that stage, and when individuals fail the treatment in that stage to progress further to the next stage. All the notations are described in the table below –

| Notations | Description |
| --- | --- |
| S_i_ | Susceptible to infection ∀ 1 ≥ i ≥ 4 |
| µ | Mortality |
| Γ | Rate at which people enter the susceptible group |
| Π_i_ | Proportion in each sexual class group ∀ 1 ≥ i ≥ 4 |
| Cl | Probability of clearance |
| τ | Time taken to clear the virus |
| Λ_Si_ | Force of infection of susceptible individuals |
| Λ_SRi_ | Force of reinfection for individuals once infected |
| SR_i_ | Susceptible to reinfection ∀ 1 ≥ i ≥ 4 |
| SVR | Probability of achieving sustained virologic response |
| n | Stage of fibrosis (0≥n≥3) or cirrhosis (1≥n≥2) |
| F | Diagnosis at Fibrosis (F0, F1, F2, F3) |
| C | Diagnosis at Cirrhosis (C1, C2) |
| FRx | Treatment at Fibrosis |
| CRx | Treatment at Cirrhosis |
| HCC | Hepatocellular carcinoma |
| ε | Factor of assortative mixing matrix ∀ 0 ≥ i ≥ 1 |
| i | Sexual risk class |
| j | Sexual risk activity |

**2.1 Ordinary differential equations for individuals not infected with HCV**

Individuals susceptible to infection are denoted by Si, where in i varies for 1 to 4 depending upon the sexual risk activity class. Susceptible individuals have a mortality rate of µ. Individuals that become sexually active enter the group of susceptible individuals at a rate of Γ times the proportion of π_i_ of each sexual activity class. Values of the parameter Γ have been calibrated to the Thai HIV positive MSM population. Individuals can clear HCV naturally with the probability of Cl multiplied to the time taken to clear the virus τ. Individuals may get reinfected to the virus after having cleared the virus or after achieving sustained virologic response (SVR), post DAA treatment. The force of infection, denoted by Λ_Si_ for those who are susceptible to HCV infection and Λ_SRi_ for those who are susceptible to reinfection of HCV, represents the rate by which people can become infected.

S_i_´ = Γπ_i_ – S_i_ (1- Cl*τ) * Λ_si_ – S_i_ (Cl*τ) - Sµ (1)

SR_i_´ = ΓCl τ + $\sum_{i=0}^{n} Fn Rx*SVRn*\delta n$ – SR_i_ (Λ_SRi_ * Cl) – SR_i_ µ (2)

Cl´ = S_i_ * (Λ_si_ Cl) + SR_i_ (Λ_SRi_ * Cl) - ΓCl τ * SR_i_ (3)

**2.2 Ordinary differential equations for individuals infected with HCV**

Individuals that become infected with HCV and have not cleared the virus or have not been treated yet, can be identified at any stage of fibrosis depending upon when they are diagnosed. The equation for the stage of fibrosis is denoted by F_n_ where n represents the stage of fibrosis from 0 to 3 progressing from acute to chronic stage. Once individuals are diagnosed and under treatment, denoted by FRx_n_, where in patients can either achieve sustained virologic response of fail treatment to progress to the next stage of the disease. Individuals can develop compensated and decompensated cirrhosis, denoted by C_n_, wherein n ranges between 1 and 2. The sustained virologic response for the DAA treatment and the duration for treatment would also vary at these stages, denoted by CRx_n_. If individuals fail the treatment at the stage of cirrhosis, they can develop hepatocellular carcinoma, denoted in the model equations by HCC.

F_n_´ ∀ 0 > n ≥ 3 = S_i_ Λ_si_ (1- Cl*τ) + SR_i_ Λ_SRi_ (1- Cl*τ) + $\sum_{n=1}^{3} {F(1-Rx)}_{n-1}{(1 - SVR)}_{n-1}*\delta_{n-1}$ $- \sum_{n=1}^{3} {F(Rx)}_{n}{(SVR)}_{n}*\delta_{n}$ $-$ F_n_µ (4)

C_n_´ ∀ 1 ≥ n ≥ 2 = S_i_ Λ_si_ (1- Cl*τ) + SR_i_ Λ_SRi_ (1- Cl*τ) + $\sum_{n=1}^{3} {F(1-Rx)}_{n-1}{(1 - SVR)}_{n-1}*\delta_{n-1}$ + $\sum_{n=1}^{2} {C(1-Rx)}_{n}{(1 - CSVR)}_{n}*{C\delta}_{n}- \sum_{n=1}^{3} {F(Rx)}_{n}{(SVR)}_{n}*\delta_{n}$ $-$ F_n_µ $-$ C_n_µ - HCC (5)

2.3 **Mixing matrix**

Individuals enter the model as per the varying degree of sexual risk group they belong to using a mixing matrix M_i,j_´ within class i and sexual activity j. ε denotes the factor of assortative mixing matrix, which takes the value between 0 and 1. It is 0 if the sexual partnership are completely random and partnerships are formed between different activity class, whereas it is 1 if the sexual partnership are completely assortative, or forming between the same sexual activity class. This mixing matrix leads to the interaction between individuals infected and not infected with Hepatitis C virus.

M_i,j_´ = εδ_i_ + (1 – ε) π_i_ $\frac{Ni}{\sum_{i=1}^{4} C_{i}\pi_{i}}$ (6)

**3. Costs of treatment**

In our analysis, we have assumed that the costs will be paid by the Thai government under the universal health insurance. Final cumulated costs were converted from Thai Baht into US dollars with a rate of conversion of 1THB = .032 USD.

| Item | Costs Range public hospitals (THB) |
| --- | --- |
| HCV RNA | 1,700 - 2,500 |
| HCV genotyping | 2,500 - 4,000 |
| Fibroscan | 2,000 - 2,500 |
| U/S liver | 800-1,500 |
| DAA regimen per month (SOF/Ledipasvir) | 13,860 |
| BUN | 40-100 |
| Creatinine | 40-100 |
| Total bili | 40-100 |
| Direct bili | 40-100 |
| ALT | 40-100 |
| AST | 40-100 |
| Alkaline phosphatase | 40-100 |
| GGT | 130-160 |
| LDH | 60-150 |
| Albumin | 50-100 |
| PT/INR | 75-100 |

|  |  |  |
| --- | --- | --- |
|  |  |  |

**4.** **Recursive Partitioning**

In the multi variate sensitivity analysis, we entered all the parameters that were varied in the model to generate the simulations, with cost-effectiveness of immediate treatment METAVIR stage F0 over base case scenario METAVIR stage F2 as the end point. Incidence rate in the year 2030 had the most dominant impact which is the primary node on explaining the variation in cost effectiveness. If it was less than 21 per 1000 person years, split occurs with proportion of individuals diagnosed at acute F0 stage of HCV explaining 27% of the variance; if the incidence rate was more than 21 per 1000 person years in 2030, then 73% of the variance was explained by the reinfection rate in 2030. Further, the nodes at reinfection rate in 2030 is split if it is above 10 per 1000 person years, in which case the variance in cost effectiveness is explained to the magnitude of 20% by proportion of individuals at the highest sexual risk category. If the reinfection rate in 2030 is less than 10 per 1000 years, then 52% of the variance is explained by individuals diagnosed at acute F0 stage of HCV.

**Figure S2:**


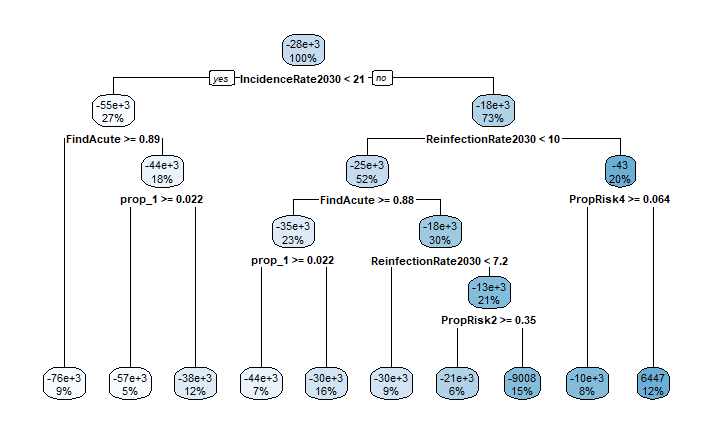

Supplement: Multimedia component 1 [file mmc1.docx]
